# Synthesis-View: visualization and interpretation of SNP association results for multi-cohort, multi-phenotype data and meta-analysis

**DOI:** 10.1186/1756-0381-3-10

**Published:** 2010-12-16

**Authors:** Sarah A Pendergrass, Scott M Dudek, Dana C Crawford, Marylyn D Ritchie

**Affiliations:** 1 Center for Human Genetics Research, Vanderbilt University Medical Center, Nashville TN, USA; 2 Department of Molecular Physiology & Biophysics, Vanderbilt University Medical Center, Nashville TN, USA

## Abstract

**Background:**

Initial genome-wide association study (GWAS) discoveries are being further explored through the use of large cohorts across multiple and diverse populations involving meta-analyses within large consortia and networks. Many of the additional studies characterize less than 100 single nucleotide polymorphisms (SNPs), often include multiple and correlated phenotypic measurements, and can include data from multiple-sites, multiple-studies, as well as multiple race/ethnicities. New approaches for visualizing resultant data are necessary in order to fully interpret results and obtain a broad view of the trends between DNA variation and phenotypes, as well as provide information on specific SNP and phenotype relationships.

**Results:**

The Synthesis-View software tool was designed to visually synthesize the results of the aforementioned types of studies. Presented herein are multiple examples of the ways Synthesis-View can be used to report results from association studies of DNA variation and phenotypes, including the visual integration of p-values or other metrics of significance, allele frequencies, sample sizes, effect size, and direction of effect.

**Conclusions:**

To truly allow a user to visually integrate multiple pieces of information typical of a genetic association study, innovative views are needed to integrate multiple pieces of information. As a result, we have created "Synthesis-View" software for the visualization of genotype-phenotype association data in multiple cohorts. Synthesis-View is freely available for non-commercial research institutions, for full details see https://chgr.mc.vanderbilt.edu/synthesisview.

## Background

Significant GWAS findings are being further investigated for replication and characterization, both in the populations in which the initial GWAS findings were discovered (such as European-Americans) as well as in new cohorts and populations. To increase power, meta-analysis is often used to combine results from multiple research sites. Multiple independent and correlated phenotypic measurements may be included in these analyses, such as measurements of cardiovascular disease and related biomarkers (lipids, inflammation, etc). Many of these studies characterize less than 100 SNPs. Visualization of these data an integral part of interpreting as well as sharing the complex and multi-layered results of these follow-up studies. The software "Synthesis-View" has been developed to visually synthesize multiple pieces of information of interest from these studies with the flexibility to perform multiple types of data comparisons.

Synthesis-View was extended from the previous software "LD-Plus" which also uses a flexible data display format of multiple data "tracks" that can be viewed [1]. Within Synthesis-View, through the use of stacked data-tracks, information on SNP genomic locations, presence of the SNP in a specific study or analysis, as well as related information such as genetic effect size and summary phenotype information, is plotted according to user preference. Through these data visualizations, rapid comparisons of multiple forms of information are possible, not easily achievable through reviewing results in tabular form alone.

## Implementation

**Figure 1:**
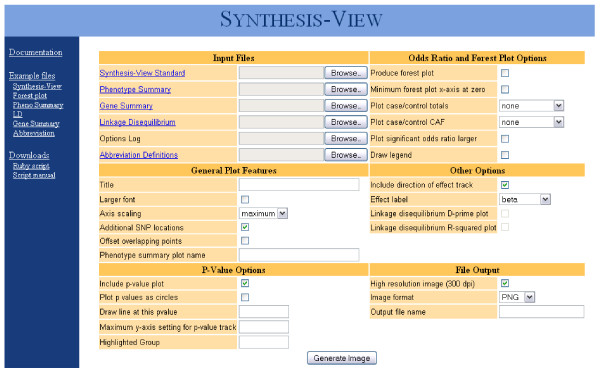
**Synthesis-View Web Interface**. Web interface for Synthesis-View. The software is also available for use at the command-line.

Synthesis-View was developed in Ruby and utilizes the RMagick graphics library, and the software tool can be used within a web-interface or at the command line. Both the web interface (Figure 1) and command line version offer multiple options for output plots (Table 1). Manuals, example files for producing test plots in Synthesis-View, and source code, are all available at the Synthesis-View website https://chgr.mc.vanderbilt.edu/synthesisview/create/setparams.

**Table 1:** Synthesis-View plotting options

Synthesis-View Option	Description
**Title**	Title for Synthesis-View plot
**Larger font**	Produce a plot with larger sized text than the default
**Axis scaling**	If set to "maximum", axes limits will start and end utilizing the range of the data with tick-marks at regular intervals in-between. If set to "cleaner" the axes will still encompass the range of the data, however the range will begin and end with a multiple of five or ten, and the plot tick-marks will also be a multiple of five or ten.
**Additional SNP locations**	SNP location information in addition to chromosomal location range.
**Offset overlapping points**	When points overlap, this setting will include "jitter", whereby overlapping points are offset horizontally to make them more distinguishable.
**Phenotype summary plot name**	If phenotypic summary data will be incorporated into the Synthesis-View plot, the title for the phenotype summary plot should be specified here.
**Include p-value plot**	Include plot of p-values
**Plot p-values as circles**	To plot p-values as circles, instead of triangles that include direction of effect, even if direction of effect information is supplied in the Synthesis-View standard input file.
**Draw line at this p-value**	Specification of a horizontal red line at a specific p-value of interest.
**Maximum y-axis setting for p-value track**	Specify the maximum y-axis value for the p-value track in order to limit the range of the y-axis. Any p-value result more significant than this y-axis cutoff value will be plotted at the cutoff value in larger size.
**Highlighted Group**	Set points as "diamond shape" for one specific group/substudy
**Produce forest plot**	To produce a forest plot in Synthesis-View from odds-ratio results
**Minimum forest plot x-axis at zero**	To set the minimum value of the forest plot x-axis to zero
**Plot case/control totals**	The total numbers of cases/controls can be plotted either in two separate tracks ("split plot"), or in one track where the total numbers of cases/controls are indicated using open/closed circles ("combined plot").
**Plot case/control CAF**	The respective coded allele frequency (CAF) for cases/controls can be plotted either as two separate tracks ("split plot"), or in one track where cases/controls are indicated using open/closed circles ("combined plot").
**Plot significant odds ratio larger**	Plot significant odds-ratio results in larger size
**Draw legend**	Draw legend for forest plot
**Include direction of effect track**	Even if direction of effect information is supplied, this setting allows for inclusion/exclusion of a direction of effect track.
**Effect label**	Choice of effect size label
**Linkage disequilibrium D-prime plot**	If linkage disequilibrium information is included as an input file, select this to include a d-prime correlation track.
**Linkage disequilibrium R-squared plot**	If linkage disequilibrium information is included as an input file, select this to include an R-squared correlation track.
**High resolution image (300 dpi)**	Select to produce a 300 dpi image, otherwise the image is 72 dpi
**Image format**	Choices of image format include PNG, JPEG, and TIFF
**Output file name**	Choice of file name for output Synthesis-View plot

One file is necessary to produce a standard Synthesis-View plot. This file contains a column for SNP identification (such as rs number), a column with the corresponding chromosome for each SNP, and a column for SNP genomic location. The rest of the optional information will result in data tracks plotted if data are present, and can include p-values, odds ratios, allele frequencies, and sample size. If additional files are supplied, additional tracks are created including phenotypic summary information for continuous phenotypes, gene summary information, and linkage disequilibrium (LD) data plotted in Haploview style [2] format as D' or r^2^.

### Availability and requirements

Project name: Synthesis-View

**Project home page**: http://chgr.mc.vanderbilt.edu/ritchielab/synthesisview

**Operating systems(s)**: Linux, Mac OS X, Windows

**Programming language**: Ruby

**Other requirements**: RMagick

**License**: GNU General Public License

Any restrictions to use by non-academics:

The use of Synthesis-View is restricted to academic and non-profit users

## Results and Discussion

### Example Standard Output Plot

**Figure 2:**
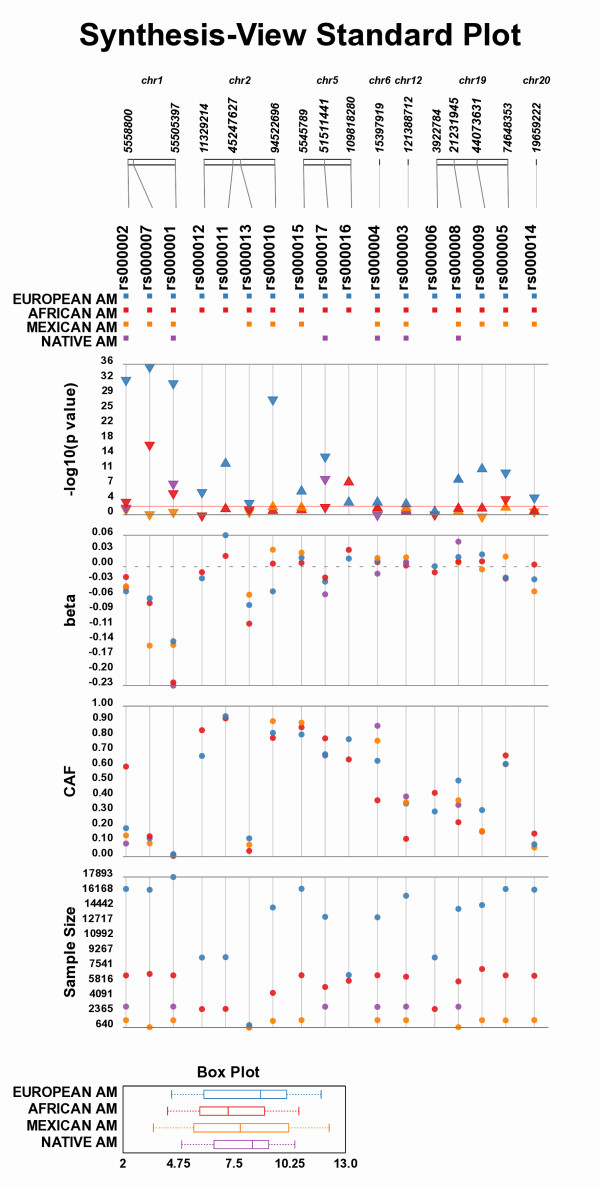
**Synthesis-View Standard Output Example Plot**. An example Synthesis-View plot generated from simulated data. Data is plotted in tracks, with various information including SNP locations, results of association tests, sample sizes, and phenotypic summary information, all in one collective image.

Figure 2 shows an example of Synthesis-View visualization for analysis across populations. For Figure 2 two input files were used (containing simulated data for example purposes): 1) containing the SNP association data and 2) containing phenotypic summary information. The various "tracks" of data are described, starting from the top to bottom:

(1) *Physical genome track*. Synthesis-View provides information on the relative location of the SNP on a given chromosome and how that position relates to other SNPs in the same study. Lines lead from the chromosome locations to the IDs of each SNP. If the "Additional SNP locations" option is selected, the location of the SNPs within the chromosomal region are indicated. If SNPs are close together, the location of the first SNP in that group is indicated in the plot (to prevent text overlap). When the plot is first generated, an image of the plot is shown within the web browser that includes embedded links. If the results for a SNP are selected within this image, the NCBI SNP database page for that specific SNP is opened in the default web browser of the user.

*(2) SNP presence/absence track*. Not all SNPs may be available for all associations across study groups or populations. Thus, this track provides information on whether a SNP was used in the test of associations through the presence/absence of a colored box corresponding to the group, study, or phenotype.

*(3) Significance track*. The resultant p-values are plotted as the negative log_10 _of the p-value. Grey vertical lines behind the points create an "abacus-view" that allows for the eye to follow from the SNP presence/absence track down through the lower data tracks. An optional red horizontal line can be applied at a significance level of choice (in Figure 2 the p-value line is set at 0.01). Figure 2 shows how the p-value for each SNP association can be compared across populations (in this case, across race/ethnicities), and triangles indicate direction of the genetic effect (bottom of the triangle is the location of the p-value). For example, if the genetic effect is measured as beta, the triangles point up if beta is positive and point down if beta is negative. The direction enables the investigator(s) to quickly determine if direction of effect is consistent across groups, studies, or phenotypes.

(4) *Effect size track*. The resultant effect size values (beta values here) are plotted. This track allows you to view the similar effect sizes across race/ethnicity in Figure 2. To omit the effect size plot, omit effect size information from the input file.

(5) *Coded allele frequency track*. The coded allele frequency (CAF), the allele chosen by the user to compare across groups or studies, is optionally plotted so trends and differences in the data can be observed.

(6) *Sample size track*. Optional plotting of the sample size for each genotype-phenotype association for each group/study/phenotype is available so the relationships between sample size and other results of the study can be explored. To plot without sample size, omit sample size information columns from the input file. If sample size is provided only as entire group summary information, rather than for individual SNP/phenotype regressions, a separate box will appear at the bottom of the plot with this summary information graphically represented.

(7) *Phenotype summary plot*. Summary information for a single phenotype across several groups is plotted if a separate file of phenotype summary information is included. This is currently a feature for quantitative traits/continuous data. Future versions will incorporate methods to characterize categorical/case-control phenotype summary information.

### Other Options for Standard Output Plot

**Figure 3:**
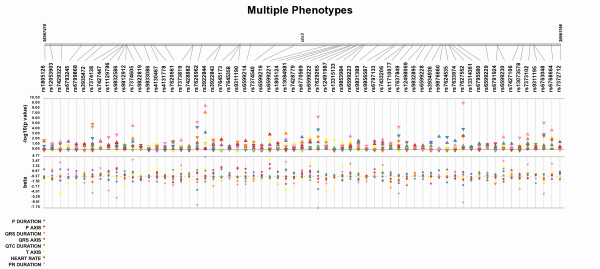
**Multiple Phenotype Synthesis-View Plot**. An example of plotting the results for tests of association, assuming an additive genetic model, for the same SNPs across multiple phenotypes (listed in the legend). Because all tests of association were available for all phenotypes, the legend on the left appeared instead of a SNP presence/absence track. (Data from Jeff et al. 2010, *in preparation*)

When all SNPs are available for all tests of association, a legend appears indicating which colors correspond to which group, study, or phenotype. Figure 3 shows an example of this feature, as well as showing an example of using Synthesis-View to plot the results for multiple phenotypes (from Jeff et al. 2010, *in preparation*). Alternate colors for the data points can be specified by adding an additional column with the header of 'Color', along with the color selected for each group, in the phenotype summary file.

**Figure 4:**
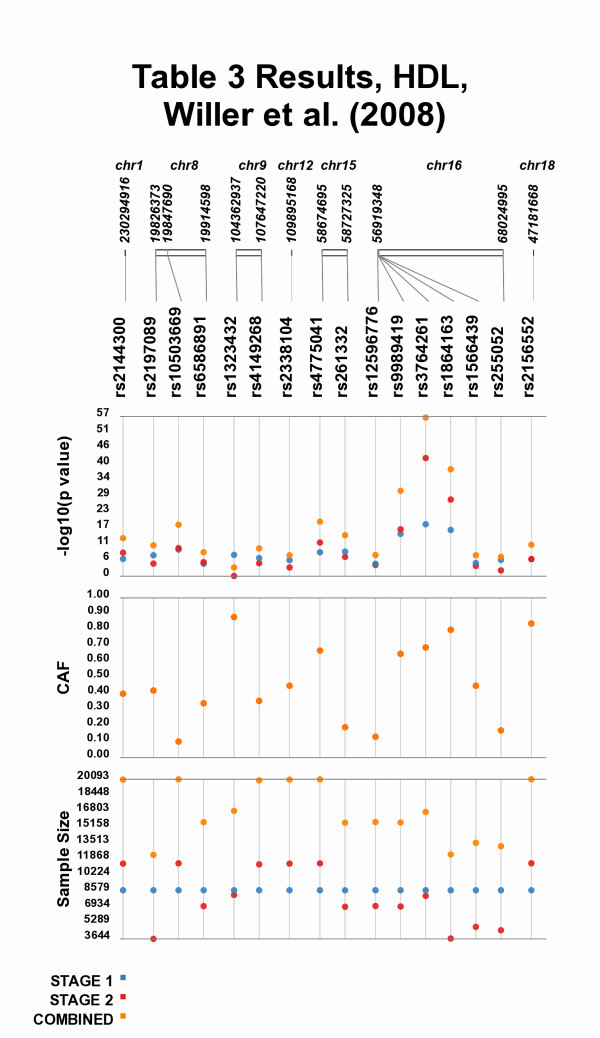
**Tabular results Moved to Visual Format**. Plotting the results from Table three, from Willer et al. 2008 [3], allowing for the visual comparison between individual stages of a study to the combined meta-analysis results.

Comparisons can also be made across study stages, such as Figure 4 where the results of Table three, from Willer et al. 2008 [3] are plotted, allowing for the visual comparison between individual stages of a study and the combined meta-analysis results. Of particular interest in Figure 4 are SNPs rs9989419, rs3764261, rs1864163, all of which have strongest association with high-density lipoprotein (HDL) cholesterol among the SNPs tested. Plotting the data in this format shows the similar location of these SNPs as well as their strong association with HDL levels. Investigators examining these data could postulate that the close proximity of these SNPs and the similar genetic effect sizes made evident by Synthesis-View suggest that these SNPs are in LD with one another, prompting further investigation of the data. Also, plotting the samples size is useful in (indirectly) visualizing the power of each test of association, which is necessary to interpret non-significant findings.

**Figure 5:**
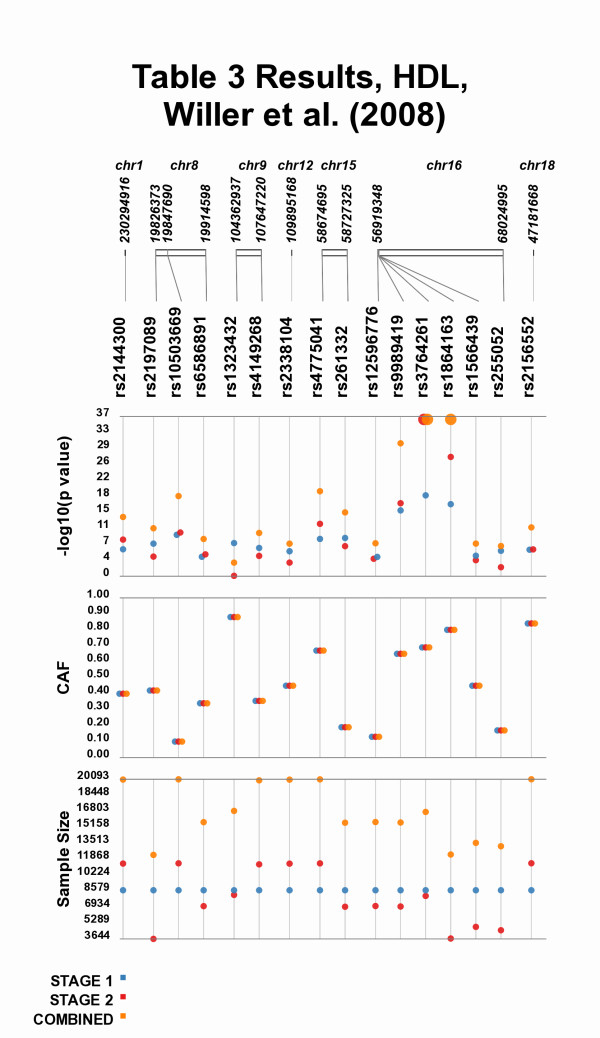
**Additional Options in Synthesis-View**. The results from Table three, from Willer et al. 2008 [3], plotted again with additional options. In this case a significance level of 1E-33 was chosen, and results more significant than that cutoff are plotted at the cutoff in larger size. Also, overlapping points are moved apart horizontally.

In Figure 5 the same Table three data from Willer et al. [3] is plotted again, however, different options are used. When there is a wide range of p-values, there can be compression of the less significant p-values when plotted, visible in Figure 4 for the results for SNPs other than rs9989419, rs3764261, rs1864163. Synthesis-View allows for the choice of significance level, such that any points more significant than the cutoff value are plotted at that value, in a larger size (in this case set at 1E-33). This feature allows for closer inspection of the less significant p-values. Also used in Figure 5 is the option for "jitter", where overlapping points are plotted with horizontal distance between them.

**Figure 6:**
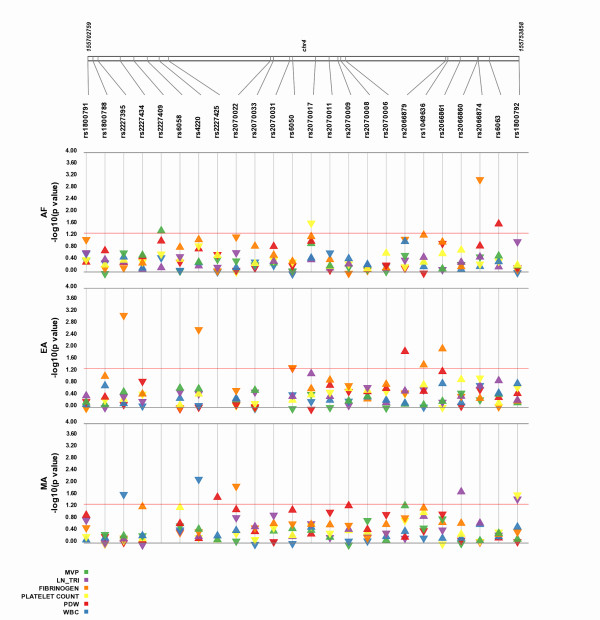
**Multiple Phenotypes and Multiple Groups**. The results for six phenotypes across three race/ethnicities (from Jeff et al, 2010, *in preparation*) are plotted. Multiple tracks are available for comparing results across multiple phenotypes from data stratified by three race/ethnicities, Mexican American (MA), European American (EA), and African American (AF).

In some settings, studies have multiple phenotypes, as well as multiple groups (such as multiple race-ethnicities). In this case, Synthesis-View will plot the results for all the phenotypes for each group on separate tracks. Figure 6 shows an example of this feature, where associations were calculated for six phenotypes and a series of SNPs (from Jeff et al. 2010 *in preparation*), and investigated for three race/ethnicities: Mexican Americans (MA); European Americans (EA); and African Americans (AF). Thus, in this plot, the results across multiple phenotypes are plotted with a track for each race/ethnicity allowing for multi-layered results to be viewed together.

**Figure 7:**
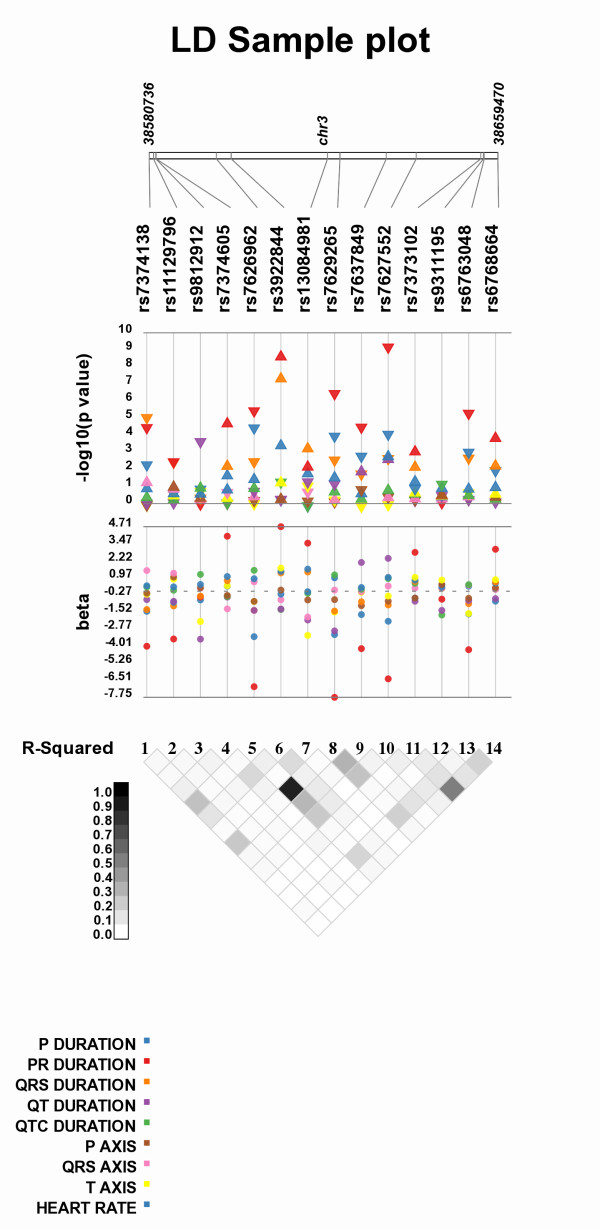
**Synthesis-View plot with r^2 ^plotted in Haploview style format**. The results for nine phenotypes (from Jeff et al, 2010, *in preparation*) are plotted. At the bottom of the image is a Synthesis-View generated r^2 ^Haploview style plot.

Another feature available in Synthesis-View is the plotting of a D' or r^2 ^plot in Haploview style format [2] as the bottom-most track, shown in Figure 7 (From Jeff et al. 2010 *in preparation*). This plot will appear when D' or r^2 ^data are provided in a separate file.

### Forest Plot/Odds Ratio options in Synthesis-View

**Figure 8:**
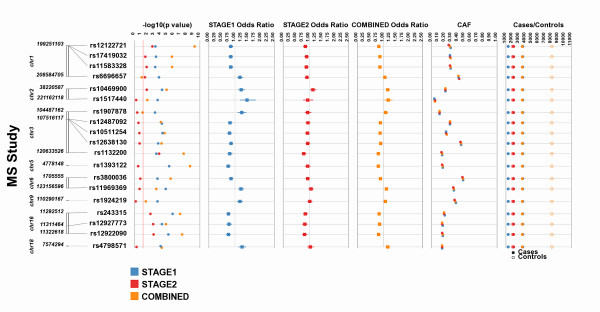
**Forest-Plot option in Synthesis-View**. Stage 1, Stage2, and the final Combined results from a study of MS by the International Multiple Sclerosis Genetics Consortium (IMSGC) were presented in Table two of manuscript [4] and the results are presented here in forest plot format using Synthesis-View.

Synthesis-View also has an option for plotting odds ratio (OR) results in forest plot format. Figure 8 shows an example from the International Multiple Sclerosis Genetics Consortium (IMSGC) [4], where original IMSGC Multiple Sclerosis GWAS results were investigated for replication in an independent dataset. A Stage 1 analysis was performed to examine the replication of a series of SNP/phenotype associations. In Stage 2 a smaller subset of 19 SNPs were tested for further replication. The results from Stage 1, Stage 2, and the final combined analysis for 19 SNPs were presented in Table two of the manuscript [4], and the results are presented here in forest plot format using Synthesis-View. The tracks are as follows:

1) The first track, like with the standard Synthesis-View plot, is a physical genome track, displaying the chromosome and relative location of each SNP used in the association tests.

2) The next track is an optional significance track, displaying the p-values. A single color represents each group. In this case, a red line has been placed at a p-value of 0.05.

3) The next three tracks are odds ratio/forest plot tracks. Squares represent the OR point estimate, with lines representing the upper and lower 95% confidence intervals. Here the similarity of the results between Stage 1 and Stage 2 are visible. An additional option, not shown, is available. If a result is significant (the upper or lower boundary of the confidence intervals does not cross 1.0), the square can be plotted in larger size, allowing for quick visual identification of significant results in forest plots with a large number of results.

4) The second to last track is the CAF track. Colors match those of the groups of the previous tracks, allowing the user to identify trends in allele frequencies between groups which can aid in interpreting replication of results. The option of horizontal separation of overlapping points was also used here as the CAF measurements were very similar between the analyses.

5) The last track is the sample size track. Case/control sample size can either be plotted in separate tracks, or, as shown here with closed circles indicating cases, and open circles indicating controls in the same track. The colors match those of the groups of the previous tracks. This option is also available when the CAF for cases vs. controls are provided.

**Figure 9:**
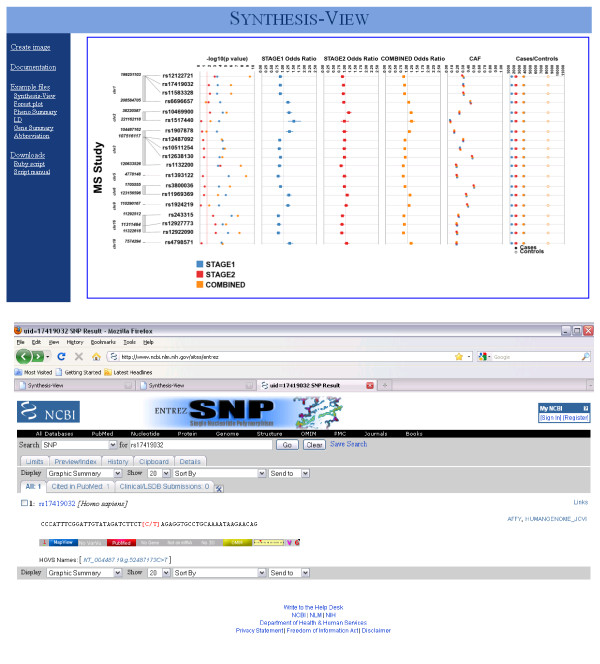
**HTML embedded image in Synthesis-View web interface**. When a plot is first generated, such shown in Figure 8, an image of the plot appears within the web browser (screen capture shown in upper panel). This plot includes embedded HTML links. When the results for a SNP are selected, the NCBI SNP Database page for that specific SNP opens in the default web browser of the user (screen capture shown in lower panel).

The upper panel of Figure 9 shows the plot that appears within the web browser, after "generate image" is selected. This plot contains embedded links for each SNP to the NCBI SNP database. The lower panel shows the NCBI SNP page that appeared when the SNP 17419032 was selected with the mouse.

An alternative way to view OR results is in stacked tracks where the eye moves from top to bottom, in more of the Synthesis-View standard format. If the forest plot option is not chosen in Synthesis-View, the default data plot is in this format. Unlike the forest plots of Figure 8 ORs are plotted as closed circles. When OR results are significant, the OR closed circle is plotted in a larger size, rendering it easy to discriminate significant results visually.

## Conclusions

To date, most replication, meta-analysis, and even top GWAS results, are presented in tabular form. There are additional ways to display these results. We developed and describe here a visualization tool for studies that have data for less than 100 SNPs, which is typical for a targeted genotyping study using thousands to tens of thousands of samples. We emphasize that Synthesis-View is especially effective when data are being investigated across phenotypes, studies, and multiple race/ethnicities. Through visually incorporating results, details of individual SNP-phenotype relationships as well as larger trends in the interplay between information such as SNP location, sample size, data stratification, and allele frequencies can be viewed.

## Competing interests

The authors declare that they have no competing interests.

## Authors' contributions

SAP carried out design of Synthesis-View, SMD programmed, DCC and MR participated in design and coordination and drafting of the manuscript. All authors read and approved the final manuscript.

## References

[B1] Bush WS Dudek SM Ritchie MD Visualizing SNP statistics in the context of linkage disequilibrium using LD-Plus Bioinformatics 2010 26 578 579 10.1093/bioinformatics/btp678 20130027 PMC2820673

[B2] Barrett JC Fry B Maller J Daly MJ Haploview: analysis and visualization of LD and haplotype maps Bioinformatics 2005 21 263 265 10.1093/bioinformatics/bth457 15297300

[B3] Willer CJ Sanna S Jackson AU Scuteri A Bonnycastle LL Clarke R Heath SC Timpson NJ Najjar SS Stringham HM Newly identified loci that influence lipid concentrations and risk of coronary artery disease Nat Genet 2008 40 161 169 10.1038/ng.76 18193043 PMC5206900

[B4] Comprehensive follow-up of the first genome-wide association study of multiple sclerosis identifies KIF21B and TMEM39A as susceptibility loci Hum Mol Genet 2010 19 953 962 10.1093/hmg/ddp542 20007504 PMC2816610

